# Editors’ Pick: mad and genius in the same gene?

**DOI:** 10.1186/2041-2223-4-14

**Published:** 2013-07-31

**Authors:** Manfred Kayser

**Affiliations:** 1 Department of Forensic Molecular Biology, Erasmus MC University Medical Centre Rotterdam, PO Box 2040, 3000, Rotterdam, CA, The Netherlands

## 

In the current world of genetic publishing and multi-author papers, where sometimes it is possible that several hundreds of co-authors are listed on a single article (and yes, I am thinking of genome-wide association studies here), I am always intrigued by single author papers. Hence, a not too recent paper by Szabolcs Kéri from Semmelweis University in Budapest drew my attention, also because of the interesting title ‘Genes for psychosis and creativity’ [1]. This reminded me of the German saying, ‘*Genie und Wahnsinn liegen oft dicht beieinander*,’ which translates as: genius and madness are often at close quarters, and is similar, but not quite the same as the English phrase, ‘There is a fine line between genius and madness.’ Does this study provide one of the not so frequent cases where a layman observation that led to commonly used phrases, even in different languages, eventually receives scientific proof?

Previous studies had suggested that the *neuregulin I* gene, which is involved in neuronal development, synaptic plasticity, glutamateric neurotransmission and glial function, serves as a candidate gene for psychosis [2]. In particular, the T/T genotype of the functional promoter polymorphism SNP8NRG243177 or rs6994992, which leads to increased *neuregulin I* gene expression, is associated with an increased risk of psychosis and other psychological and neurological phenotypes [3,4]. Kéri investigated whether this particular DNA variant also has an advantageous effect. He studied 200 healthy volunteers with high intellectual performance as measured via a battery of tests and questionnaires, who were genotyped at the *neuregulin 1* promoter DNA variant rs6994992. Grouping these 200 high intellectual achievers according to rs6994992 genotypes revealed that the T/T carriers had statistically significantly higher creativity measures compared with the C/C and the C/T carriers, with the C/T heterozygotes usually performing at an intermediate level relative to the T/T and the C/C homozygotes. No significant differences between individuals belonging to these three genotype groups were observed for other parameters tested, such as age, gender, education, socioeconomic status, intelligence quotient (IQ), employment, marriage, and so on. Two additional DNA variants from the *neuregulin I* gene that are not implicated in psychosis and do not affect gene expression were also tested and did not indicate similar associations. Based on these data, Kéri argues that the *neuregulin I* gene, previously implicated in psychosis and altered brain structure and function, has a significant impact on human creativity.

Is this evidence enough to support the conclusion that genius and madness are indeed related to each other because they are determined by the same gene? The author himself already admits some caveats in his study, such as that the findings were obtained from a selected group of people with high intellectual performance. It may be (as was not tested) that the observed effect is not found in an intellectually less-prominent sample. He also points out that in the general population rs6994992 is not associated with schizotypical traits as reported previously [5]. Furthermore, genetic evidence on the involvement of *neuregulin 1* in psychotic conditions appears not without doubt. For instance, while some candidate gene studies reported the association of *neuregulin-1* DNA variants with schizophrenia (for example [6]) and a genome-wide linkage analysis highlighted a candidate region for schizophrenia that includes the *neuregulin-1* gene [7], several genome-wide association studies highlighted genes other than *neuregulin-1* to be involved in schizophrenia (for example [8,9]).

If Kéri’s findings indeed reflect a general trend and if, indeed, the *neuregulin 1* gene determines psychotic conditions, the question on how the rs6994992T/T genotype leads to higher creativity will be of interest. The author suggests that reduced cognitive inhibition, known to be related to schizotypical features and associated with increased creativity in people with high intelligence, may be the key here, but data on the direct role of rs6994992 in reduced cognitive inhibition is missing thus far.

Clearly, much further work needs to be done before scientific data allow us to conclude whether madness and genius indeed share biological determination and, thus, can be considered to be at close quarters, or not. Until this is achieved, we may keep using the saying ‘*Genie und Wahnsinn liegen oft dicht beieinander*,’ but need to be aware that scientific evidence clearly supporting it is still lacking.

## Competing interests

The author declares that he have no competing interests.
